# Prevalence of Dental Caries and Its Associated Factors Among Diabetic Patients at Mbarara Regional Referral Hospital

**DOI:** 10.7759/cureus.93556

**Published:** 2025-09-30

**Authors:** Amon Mwesigwa, Evas Nimusiima, Godfrey Kwizera, Edgar M Mulogo

**Affiliations:** 1 Department of Dentistry, Brighter Smiles Dental Services, Mbarara, UGA; 2 Department of Dentistry, Mbarara Regional Referral Hospital, Mbarara, UGA; 3 Department of Community Health, Mbarara University of Science and Technology, Mbarara, UGA; 4 Department of Dental Surgery, Mbarara University of Science and Technology, Mbarara, UGA

**Keywords:** dental caries, factors associated with dental caries, oral diseases, prevalence of dental caries, type 2 diabetes

## Abstract

Introduction: Dental caries continues to affect oral health quality of life among diabetic patients. However, there is limited data on the prevalence and factors associated with dental caries among diabetic patients in Uganda. Therefore, this study assessed the prevalence and factors associated with dental caries among diabetic patients attending a diabetic clinic at a regional referral hospital in Uganda.

Method: A clinical cross-sectional study was conducted among 336 diabetic patients attending a diabetic clinic at Mbarara regional referral hospital from October to December 2023. A consecutive sampling technique was employed to enroll study participants. Data were collected using a pretested structured questionnaire, and analysis was performed using STATA version 17. Bivariable and multivariable logistic regressions were employed and variables with a p-value < 0.05 were declared statistically significant. The prevalence of dental caries was determined based DMFT index score and presented as a percentage.

Results: The prevalence of dental caries was 72.9% (95%CI: 67.9, 77.4). Findings indicated that age >57years (AOR 2.131, 95% CI 1.192-3.809 P-Value 0.011), smoking (AOR 2.884, 95%CI 1.032-8.061, p0.043) and type 2 diabetes (AOR 0. 513, 95% CI 0.272-0.966, p0.039) and visiting a dental clinic once a year (AOR 3.198, 95%CI 1.709-5.982, p<0.001) were associated with increased odds of developing dental caries. Brushing twice a day (OR 0.394, 95%CI 0.158-0.981, p 0.045) was associated with reduced odds of developing dental caries.

Conclusion. The prevalence of dental caries among diabetic patients is relatively high; this contributes significantly to their reduced oral health quality of life. Therefore, oral health promotion, preventive and curative services should be integrated in diabetic care to improve oral health and diabetic quality of life.

## Introduction

Diabetes mellitus is a metabolic disease characterized by hyperglycemia due to defects in insulin secretion, action, or both [1,2]. It is estimated that type 2 diabetes affects approximately 462 million people worldwide, with projections to rise to 7,079 per 100,000 by 2030 and 7,862 by 2040. In 2017, more than one million mortalities due to diabetes were recorded, making it one of the leading causes of death worldwide [3].

Diabetes affects oral cavity structures and there are various diseases associated with diabetes. A recent systematic review revealed that more than 90% of people with diabetes were identified as having oral disorders [4]. These oral disorders related to diabetes include xerostomia, burning mouth, gingivitis, periodontitis, tooth decay, and oral candidiasis, among others [5,6]. The risk of these oral complications is higher among patients with no glycemic control [7]. In diabetic patients, decreased salivary secretion leads to decreased cleaning and buffering capacity, in addition to decreased calcium levels that promote oral dental complications [8].

Among all oral complications, tooth decay is typical among diabetic patients. A majority of 73.33% of the Indian type 2 diabetes mellitus (T2DM) patients were found to have dental caries [9], and 89% of diabetics in Pakistan were at high risk of tooth decay [10]. The high manifestation of oral complications among T2DM patients implies that dental caries is a major concern among these patients [4]. Evidence shows that dental caries significantly affects the emotional, psychological, and physical well-being of diabetic patients [11-13]. More so, dental caries leads to cellulites, dental abscesses, and Ludwig’s angina. These make it hard to achieve optimal blood sugar control and in turn lead to other systemic complications such as diabetic nephropathy, worsening the well-being of diabetic patients [11-13].

In 2021, the Uganda Ministry of Health reported an upsurge in the number of people diagnosed with diabetes as compared to previous years [14]. However, literature regarding the prevalence of dental caries and associated factors among diabetic patients remains scanty, especially in southwestern Uganda. Therefore, this study was conducted to determine the prevalence and factors associated with dental caries among diabetic patients at Mbarara Regional Referral Hospital in southwestern Uganda.

## Materials and methods

Study design and setting

This was a cross-sectional study design that employed quantitative research methods to determine the prevalence of dental caries and associated factors among diabetic patients. 

This study was conducted at the diabetic clinic of Mbarara Regional Referral Hospital (MRRH) from October to December 2023. Mbarara Regional Referral Hospital is a government facility for the south-western region of Uganda and doubles as a teaching hospital for Mbarara University of Science and Technology. MRRH serves the whole of southwestern Uganda but also receives patients from the Central region of Uganda and neighboring East African countries, especially Rwanda, Tanzania, and the Democratic Republic of Congo. This facility operates an outpatient diabetic clinic with about 400 diabetic patients attending the clinic quarterly. The facility also enrolls about 40 new cases of diabetes quarterly.

Study participants and sampling

Adult diabetic patients who reported to the diabetic clinic of Mbarara Regional Referral Hospital participated in this study.

The study had a sample size of 336 respondents. This was determined using the Kish & Leslie formula of 1965; n= Z^2^ p q / d^2, ^which is commonly applied for prevalence and cross-sectional studies [15]. We used a Z score of 1.96, prevalence of 0.323 as obtained from a case control study in which participants were examined according to World Health Organization (WHO) criteria [16], a q= 0.677 and a standard error of 5% d=0.05. The consecutive sampling method was used to obtain the sample size.

Inclusion Criteria

The study included participants aged 18 years or above who had been diagnosed with diabetes for the past six months or above and consented to participate in the study.

Exclusion Criteria

This study excluded participants who had diabetic emergencies such as diabetic ketoacidosis, hypoglycemia, unconsciousness at MRRH, and those who did not meet this study's inclusion criteria.

All respondents underwent clinical examination to assess for dental caries, and an interviewer-administered questionnaire was used to obtain demographic data and assess for associated factors. Each day of data collection, the PI checked the diabetes register to identify patients who were present that day and had never been recruited in this study. Eligible participants were given information regarding the nature of the study, and written informed consent was obtained in a local language (Runyankore) from all enrolled participants by a trained research assistant. 

Ethical consideration

Ethical clearance was sought from Mbarara University of Science and Technology (MUST) - Research Ethics Committee (MUST-2023-829) and MRRH administrative permission was sought from the Hospital Director. Informed consent was obtained from all the recruited participants by signing or inserting a thumbprint on the informed consent form before they were recruited. Participants enrolled were free and could voluntarily withdraw from the study at any time. Confidentiality was ensured through unique identifiers rather than names and participants were examined and interviewed in a separate room with only the research team. Collected research data was kept under lock and key, only accessible by the study team with authorization from the study Principal Investigator.

Data collection tools

An interviewer-administered questionnaire, as shown in Appendix 1, was developed after a review of relevant literature from previous studies. The questionnaire consisted of three sections, A, B, and C. Closed-ended questions were used for all sections. Section A consisted of items about social demographic characteristics: Age (<=57, >57), sex, level of education, income level (<=2USD vs. > 2USD), and marital status. Section B consisted of items measuring the prevalence of dental caries. Individual clinical assessments were done using mouth mirrors under natural lighting conditions to ascertain the prevalence of dental caries. We adapted an open-access World Health Organization (WHO) scoring tool for the Decayed, Missing, Filled Teeth (DMFT) index [17]. Section C consisted of items on factors associated with dental caries: history of smoking, alcohol use, knowledge about oral diseases, type of diabetes, glycemic control, frequency of brushing, and frequency of dental checkups. The questionnaire was pretested on six diabetic patients attending the diabetic clinic at Mbarara City Health Center IV to test for consistency and predict accuracy and align its interpretation. The findings of the pretest were used to improve consistency, sequence, and shortening of the questionnaire. Being diabetic was measured as: First in sugar >7.1 mmol/L, Random >11.1 mmol/L, and HbA1c > 6.5 mmol/L. glycemic control was measured as controlled blood sugars (HbA1c 4-6.5 mmol/L) and uncontrolled blood sugars (HbA1c >6.5 mmol/L).

Data management and analysis

The prevalence of dental caries among diabetes patients was the number of diabetic patients with dental caries based on the DMFT score expressed as a fraction of the total number of diabetic patients recruited and presented with a 95% confidence interval (CI).

Bivariate and multivariate logistic regression were done to assess the association between the independent factors and dental caries. Variables that were significant P< 0.2 at 95% confidence interval at bivariate analysis were included in the multivariate model. Further variables that were not significant from the bivariate analysis but had biological and social plausibility in relation to the outcome were included in the model. Significance was set at P value <= 0.05. Adjusted odds ratios (AORs) and 95% CIs are reported.

## Results

Participant’s demographic characteristics

A total of 336 participants at Mbarara Regional Referral Hospital diabetic clinic were interviewed and screened from July to September 2023. All participants were diabetic and receiving treatment and care at the MRRH diabetic clinic. Table 1 presents the general characteristics of the samples.

**Table 1:** Demographic characteristics of study participants SD: Standard Deviation; USD: United States Dollars

Variable	Frequency N=336 (%)
Age in years (Mean, SD)	57.2 (12.9)
<= 57	177 (52.7)
>57	159 (47.3)
Sex	
Male	111 (33)
Female	225 (67)
Marital Status	
Single	13 (3.9)
Married/cohabiting	226 (67.3)
Divorced	18 (5.4)
Widowed	79 (23.5)
Level of Education	
No formal education	85 (25.3)
Elementary school	153 (45.5)
High school	68 (20.2)
Tertiary	30 (8.9)
Income level	
<= 2 USD per day	188 (56.8)
>2 USD per day	143 (43.2)
Glycemic Control	
Controlled sugar levels	264 (79)
Uncontrolled sugar levels	70 (21)

The population mean age was 57.2 (±12.9) and 52.7% of them were aged below 57 years old. 67% of the participants were female, and 67.3% had families either officially married or cohabiting. 45.5% of the participants had attained elementary class while 25.3% and 20.2% of the participants had no formal education and high school, respectively. 56.8% of participants were earning less than $2 per day and 79% of participants had controlled sugar levels.

Prevalence of dental caries among diabetic patients at MRRH

Overall, the prevalence of dental caries based on decayed, missing and filled teeth (DMFT) was 72.9% with a mean score of 4.19 (SD 4.828). As shown in Table 2, over 60% of the participants had decayed teeth, over 57% had missing teeth and 14% had filled teeth.

**Table 2:** DMFT scores of study participants DMFT: Decayed, Missing, and Filled teeth; Std.: Standard Deviation The table shows frequency distribution of decayed, missing and filled teeth of study participants.

DMFT score	Frequency N =336(%)	Mean	Std.
Decayed teeth	204 (60.7)	1.753	2.144
Missing teeth	190 (56.5)	2.152	3.369
Filled teeth	47 (14.0)	0.286	0.902
Dental caries experience (DMFT)	245 (72.9)	4.190	4.828

Factors associated with dental caries among diabetic patients

Table 3 shows the factors associated with dental caries at bivariate and multivariate levels. Results showed that participants aged above 57 years had high odds of developing dental caries at bivariate analysis (OR 2.586, 95% CI 1.6-4.3, p<0.001). Similar findings were statistically significant at the multivariate level (AOR 2.114, 95% CI 1.2-3.8, P=0.012). Participants with type 2 diabetes had odds of developing dental caries at bivariate analysis (OR 0.549, 95%CI 0.3-0.9, P=0.040). This finding was statistically significant at multivariate analysis (AOR 0. 511, 95% CI 0.3-1.0, P=0.038). Participants who smoked were 3.8 times more likely to develop dental caries compared to their counterparts at the bivariate level (OR 3.784, 95%CI 1.5-9.9, P=0.007). This finding was statistically significant at multivariate analysis (AOR 2.737, 95%CI 0.9-7.9, P=0.063). Participants who visited a dental clinic once a year had higher odds of developing dental caries as compared to their counterparts at the bivariate level (OR 2.583, 95%CI 1.5-4.5, P=0.001). Similar findings were statistically significant at multivariate analysis (AOR 3.196, 95%CI 1.7-6.0, P<0.001). Brushing your teeth twice a day was found to be associated with lower odds of developing dental caries at the bivariate level (OR 0.394, 95%CI 0.2-1.0, P=0.045). However, the protective effect did not remain significant after adjustment.

**Table 3:** Bivariate and multivariate analysis of factors associated with dental caries * statistical significance at bi-variate analysis at P-value <0.2 ** statistical significance at multivariate analysis at P-value <0.05 AOR: Adjusted Odds Ratio; COR: Crude Odds Ratio; USD: United States Dollar The table shows bivariate and multivariate analysis of factors associated with dental caries among diabetic patients at Mbarara Regional Referral Hospital.

Factors	Bivariate analysis				Multivariate analysis		
	No Dental Caries n (%)	Dental Caries n (%)	COR	P-value	AOR	P-value	
							Age in years							
<= 57	63 (35.59)	114 (64.41)	1				
> 57	28 (17.61)	131 (82.39)	2.586 (1.6-4.3)	<0.001*	2.114(1.2-3.8)	0.012**	
Sex							
Male	23 (20.72)	88 (79.28)	1				
Female	68 (30.22)	157 (69.78)	0.603(0.4-1.0)	0.067*	0.887(0.4-1.7)	0.707	
Marital Status							
Single	4(4.40)	9(3.37)	1				
Married	60(65.93)	166(67.76)	1.229 (0.4-4.1)	0.739			
Divorced	7(7.69)	11(4.49)	0.698 (0.2-3.2)	0.642			
Widow	20(21.98)	59(24.08)	1.311 (0.4-7.3)	0.679			
Income Level							
<=2USD per day	55 (29.26)	133 (70.74)	1				
>2USD per day	34 (23.78)	109 (76.22)	1.326 (0.8-2.1)	0.266			
Knowledge of oral disease							
No	27 (26.21)	76 (73.79)	1				
Yes	62 (27.19)	166 (72.81)	0.951(0.6-1.6)	0.852			
Diabetes type							
Type1	19 (19.19)	80 (80.81)	1		1		
Type2	71 (30.21)	164 (69.79)	0.549 (0.3-0.9)	0.040*	0.511(0.3-1.0)	0.038**	
Oral hygiene							
No brushing	7 (17.07)	34 (82.93)	1		1		
Once a day	50 (25.64)	145 (74.36)	0.597 (0.3-1.4)	0.248	0.704(0.3-1.9)	0.491	
Twice a day	34 (34.34)	65 (65.66)	0.394 (0.2-1.0)	0.045*	0.664(0.2-1.9)	0.449	
Smoke							
No	86 (30.07)	200 (69.93)	1				
Yes	44 (89.80)	5 (10.20)	3.784(1.5-9.9)	0.007*	2.737(0.9-7.9)	0.063**	
Freq. of dental visits							
Never	61 (34.66)	115 (65.34)	1				
Once a year	23 (17.04)	112 (82.96)	2.583 (1.5-4.5)	0.001*	3.196(1.7-6.0)	<0.001**	
Twice a year	6 (30.00)	14 (70.00)	1.238 (0.5-3.4)	0.678	1.153(0.4-3.6)	0.805	
More than thrice a year	1 (20.00)	4 (80.00)	2.122 (0.2-3.4)	0.505	0.808(0.1-9.8)	0.867	
Use alcohol							
No	88 (28.39)	222 (71.61)	1				
Yes	3 (11.54)	23 (88.46)	3.039(0.9-10.3)	0.076*	2.610(0.7-9.8)	0.130	

## Discussion

The prevalence of dental caries was high, which is similar to a previous prevalence from a study conducted on the same study site among participants of similar characteristics [18]. Also, a study conducted by Shiferaw et al. [19] found that the prevalence of dental caries was 79.6% in diabetics and 67.9% in non-diabetics, which is statistically significant. This demonstrates that being diabetic increases one's odds of developing dental caries, which is also supported by Shiferaw et al. [19] and López-López et al. [20]. This could be associated with a higher content of salivary glucose in diabetic patients, age, and a lack of awareness of oral disease among diabetic patients.

A study done in Uganda in 2023 found that the prevalence of dental caries among diabetic patients was only statistically significant in widows [18]. This finding contrasts with the findings of our study, where marital status was not associated with the development of dental caries. However, in both studies, increasing age was found to be statistically associated with the development of dental caries. Advancing in age comes along with a decreased immune system and disruption of tissue integrity, which increases susceptibility to oral diseases, especially dental caries [21].

Being above 57 years of age, having type 2 diabetes, smoking, and visiting a dental clinic once a year were significantly associated with dental caries. On the other hand, participants who brushed twice a day had reduced chances of developing dental caries. A study done by Geleto et al. [22] on dental caries and associated factors among patients visiting Shashamane Comprehensive Specialized Hospital found oral hygiene to be significantly associated with dental caries in their study. This is similar to the findings of our study. However, the other findings on income level and age contrast with those of our study. Also, a study done by Teshome et al. [23] found that participants who had poor oral hygiene had a high prevalence of developing dental caries. This is congruent with our study findings. Poor oral hygiene increases streptococcus bacteria, which are harmful to the enamel by demineralizing it. This eventually leads to dental caries [24]. More so, a study conducted by Australian adults found that the overall mean of decayed tooth surfaces was 3 times greater in adults who made less frequent dental visits as compared to their counterparts who made more frequent dental visits [25]. This finding is congruent with our study findings, where participants who made a dental visit once a year were more likely to have dental caries as compared to participants who visited more than once a year.

Though sex was not significantly associated with dental caries in this study, females had reduced chances of developing dental caries compared to males. This, however, contrasts with the results from a study done in North West Ethiopia, which found that females were 2.15 times at risk of developing dental caries [23]. Previous studies elaborate that female children have an early eruption of teeth, and so they get easily exposed to cariogenic foods [26]. There is no clear scientific literature that explains why adult males are more at a risk of dental caries than their counterparts are.

This study was limited by the cross-sectional study design and the consecutive sampling methodology employed. Therefore, the causal relationship could not be established, but also consecutive sampling could have introduced selection bias affecting the representativeness of the study sample and generalizability of study findings. More so, the study did not explore potential confounders like diet, history of medication, and use of fluoride by participants, which can influence the occurrence of dental caries. Assessment of caries under natural lighting conditions using mirrors and probes without radiographs could have underestimated the prevalence of dental caries. However, this study enrolled participants from one regional referral hospital whose profiles are similar to diabetic patients in other lower facilities. Therefore, our findings make an important contribution towards understanding the burden of dental caries among all diabetic patients in southwestern Uganda.

## Conclusions

This study shows a high prevalence of dental caries among diabetic patients and demonstrates a strong association between dental caries and increasing age, oral hygiene, conducting a dental visit once a year, type 2 diabetes, and smoking. These findings provide a basis that calls for integration of oral health services into the non-communicable disease healthcare package, especially in diabetes care. Ensuring that health professionals are aware of the burden of dental caries is also key. It allows them to appropriately participate, lobby, and link patients to dental health professionals for oral disease prevention.

## Figures and Tables

**Figure 1:**
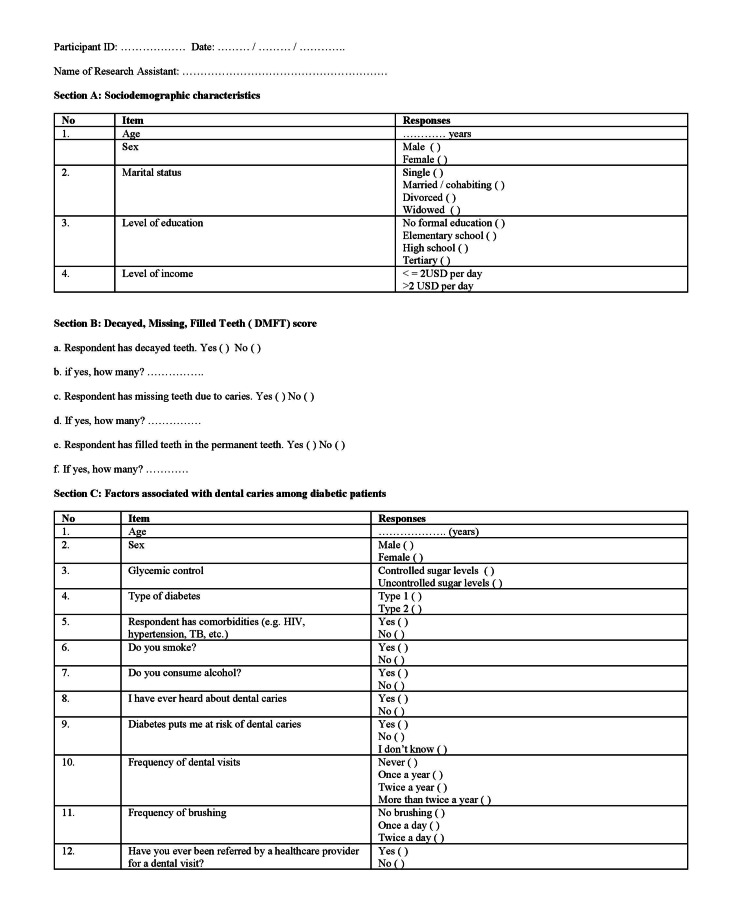
Data collection tool/questionnaire USD: United States Dollars

## Appendices

Appendix 1: Data collection tool 
